# Household healthcare seeking patterns for emergent ill-health in Migori, Western Kenya

**DOI:** 10.3389/fpubh.2025.1658217

**Published:** 2025-11-10

**Authors:** Zachary A. Kwena, Elizabeth A. Bukusi, Chanda Mwamba, Norton M. Sang, Felix O. Okoth, Jennifer F. Morton, Anna Winters, Derek Pollard, Alastair Van Heerden, Hilton Humphries, Dino Rech, Meagan Bemer, Shawna Cooper, Anjali Sharma, Paul K. Drain

**Affiliations:** 1 Centre for Microbiology Research, Kenya Medical Research Institute, Nairobi, Kenya; 2 Centre for Infectious Disease Research in Zambia, Lusaka, Zambia; 3 Department of Global Health, University of Washington, Seattle, WA, United States; 4 Akros, Lusaka, Zambia; 5 Human Sciences Research Council (HSRC), Pretoria, South Africa; 6 Audere, Johannesburg, South Africa; 7 Audere, Seattle, WA, United States; 8 School of Medicine, University of Washington, Seattle, WA, United States

**Keywords:** healthcare decision-making, emergent ill-health, healthcare-seeking behavior, access to health facilities, psycho-social factors, enabling factors, need factors, Western Kenya

## Abstract

**Background:**

Healthcare-seeking decisions reflect personal assessments of illness etiology and severity to determine whether, how, and where to seek care. Understanding these contexts enables targeted interventions to address barriers and ensure timely access to quality services. We examined patterns of household healthcare seeking for emergent ill-health in Western Kenya.

**Methods:**

As part of a multi-site mixed-methods study evaluating a patient-centered, digitally-facilitated self-testing intervention, we analyzed 16 in-depth interviews and two focus group discussions (*n* = 14) in Migori County, Kenya. Guided by the Andersen Expanded Behavioral Model, we explored psychosocial, enabling, and need factors influencing household decisions.

**Results:**

We found that a complex interplay of psychosocial, enabling, and need factors determined if, when, and where households sought care for ill members. Psychosocial influences included prevailing community norms that favored home remedies and self-medication prior to formal care as in the quote below: ‘*we will start with home remedy, then to self-medication, then we will start looking for those healers…*'. Enabling factors, particularly long waiting times, poor service quality, and lack of transport, discouraged timely facility use, leading households to rely on local pharmacies or alternative providers as reported by this participant ‘*When you come here [hospital], you will stay for too long, and then… you will be sent to the chemist to buy medication. So, I am taking the cheaper route, I just go to the chemist [pharmacy] direct I take the medicine…*' Distance to facilities, financial barriers, and lack of health insurance compromise care-seeking. Need-related considerations, especially perceived illness severity, attribution of cause, and the identity of the affected household member (with children prioritized), determined the urgency and type of care sought as illustrated in the quote ‘*There are some infections that you do not go to the hospital and some that you have to go to the hospital. So, it depends on the disease you have*'.

**Conclusion:**

Household healthcare-seeking decisions were shaped by a dynamic interplay of psychosocial, enabling, and need factors. Addressing these multifaceted barriers through targeted, context-specific interventions, such as enhancing health infrastructure and integrating different providers, is crucial to ensuring timely access to quality healthcare.

## Background

Healthcare-seeking behavior (HSB), defined as any activity undertaken by individuals who perceive themselves to have a health-related problem with the aim of finding an appropriate remedy, is critical for better health outcomes (1). Intra-household dynamics play a crucial role in the timely utilization of healthcare services for household members, especially children (2). The households' healthcare-seeking behavior involves a series of decision-making processes that include assessing the etiology (natural vs. supernatural) and the seriousness of the illness (3, 4) that inform decisions on whether to seek treatment, when and from whom to seek treatment, what kind of treatment to seek, as well as what resources are available (5–7). Thus, a household member's healthcare needs do not directly turn into effective demand for health services but go through a series of appraisals before an effective demand is finalized.

More than half of the illnesses that households encounter, such as malaria, pneumonia, diarrhea, and measles, are preventable and treatable if management decisions are made in time (8, 9). Even though the recommended point of care and treatment, especially for childhood illnesses, is a health facility (10), poor healthcare-seeking behaviors persist in sub-Saharan Africa that are informed by accessibility factors, including proximity and cost. In the face of inadequate healthcare facilities and biting staffing challenges, a big dilemma has always been how to ensure equitable access to healthcare services (11, 12). Inequitable access has resulted in the use of suboptimal healthcare alternatives, such as self-medication and traditional supernatural interventions, that delay allopathic interventions. Many factors, including personal, interpersonal, health system, structural, and policy, are known to affect households' healthcare-seeking behaviors (13–16). For instance, some sociodemographic factors such as education, age, and gender of the heads of the household are known to affect healthcare-seeking behavior (16). Additionally, the healthcare-seeking behavior has been observed to be better in extended families, where members can substitute for female caregivers who may be occupied by work or other engagements (17).

Although interventions aim to enhance health infrastructure and capacity, their success depends on the active participation of the intended beneficiaries. For instance, if households do not make the right decisions to send members for care when indicated, the health facilities with all the capacity become dysfunctional and unlikely to achieve their health goals (16, 18). Time taken between the onset of symptoms and access to allopathic care is critical in the acute management of diseases (11). Delay in diagnosis and initiation of treatment, especially for childhood pneumonia and infectious diseases such as TB, may increase infectivity, worsen the disease state, and enhance the risk of death (19, 20). For diverse reasons, people may waste precious time trying home interventions without a proper diagnosis of their conditions (21). Understanding healthcare-seeking patterns within households and their reasons is critical in finding interventions that minimize the time to receive specialized care.

To date, few studies have addressed household healthcare-seeking patterns, particularly regarding emergent and chronic conditions. Understanding factors influencing household healthcare-seeking decisions allows targeted interventions to address barriers and ensure timely access to quality healthcare for improved health outcomes. Theoretical frameworks such as the Andersen Expanded Behavioral Model of Health Services Use (22–24) have provided context for explaining the processes and considerations that come into play when a household member falls sick. Andersen's model explains healthcare utilization based on the household's psychosocial, enabling, and need factors (25). Using the constructs of psychosocial, enabling, and need factors under this framework, we sought to examine patterns of household healthcare seeking for emergent ill-health in southwestern Kenya.

## Methods

### Design

This qualitative analysis was part of a multi-site mixed methods study to evaluate the acceptability and feasibility of a patient-centered, digitally facilitated integrated healthcare intervention for addressing acute infectious and chronic non-communicable diseases in rural and urban communities in Kenya, South Africa, and Zambia. Using qualitative methods, the Kenyan site sought to assess healthcare-seeking patterns and healthcare access utilization in-depth interviews and focus group discussions.

### Study population and settings

This study was conducted in rural, peri-urban, and urban areas of Migori County and targeted community members in different settings, including fishing, mining, and farming. Our population also included healthcare providers and managers, as well as people at policy-making levels. Migori County is one of the 47 counties of Kenya, bordering Lake Victoria in the southwest of the country. It has an estimated population of 1,116,436, comprising 536,187 males and 580,214 females, as per the 2019 National Census (26). Administratively, the county is divided into 10 sub-counties, each further divided into wards, totaling 40. With an average household size of 4.6 and a growth rate of 3.1%, the county is one of the most densely populated, with an average of 427 persons/km^2^. About 90% of the population lives in rural areas, with agriculture and fishing as the primary sources of livelihood (27, 28). While the county has no referral hospital, it has 11 sub-county hospitals, 20 health centers, and 95 dispensaries (29). The county has an average life expectancy at birth of 55.5 years, with females having a relatively higher expectancy (60.6 years) compared to males (50.5 years) (30). Among the observed healthcare access challenges are poor roads and an inadequate skilled staff to operate the modern equipment.

### Sample size and sampling procedure

We conducted 16 in-depth interviews with community members and two focus group discussions with 14 community members. One FGD was reserved for women aged 25–45, and the second one consisted of young males aged 18–24. We purposefully sampled information-rich participants representing different economic activities in which they were involved (e.g., farming, mining, fishing), cadre/position in the communities (e.g., teachers, healthcare providers, religious leaders), and geographic areas (e.g., rural, peri-urban, and urban). This approach allowed us to capture a broad range of perspectives, ensuring that the voices of different socio-economic and occupational groups were represented in the study.

### Data collection

Research Assistants (RAs) worked with identified participants to schedule interviews and focus group discussions at a time and venue convenient to participants, ensuring privacy. We invited participants to community social venues, health facilities, and other conducive venues for interviews. Participants arriving at the venue were directed to a private room for consenting before participating in the in-depth interview or FGD. FGD participants were assigned numbers for use during the discussion instead of their names for confidentiality purposes. The FGD and IDI guides explored issues around the patient experience or health journey, including healthcare-seeking decision-making processes, healthcare utilization, and access for emergent and chronic diseases such as HIV and diabetes. Other topics covered included the healthcare system, provider roles, and their activities at the facilities. The discussions and interviews were audio-recorded and took an average of 2 h and 1 h, respectively. The interviews and discussions were conducted either in English, Kiswahili, or Dholuo, depending on participant preference.

### Data analysis

The audio files from the discussions and interviews were transcribed into English, de-identified, and stored securely in a password-protected folder. To ensure transcription quality, a different study staff validated the accuracy of the transcript by directly comparing part of it with the audio file. Any changes needed were discussed and made jointly with the RA who conducted the transcription. We applied deductive and inductive approaches to develop a codebook based on themes in the guide and the insights from the transcripts when they became available. The codebook and transcripts were then imported into Nvivo for coding. To achieve inter-coder reliability, the coding team of three, led by AS, coded two transcripts jointly and discussed the differences in the application of codes to build consensus and consistency in code application. The remaining transcripts were shared among three coders for individual coding. After coding, the code reports were generated for framework analysis that explored households' perspectives on healthcare-seeking decision-making and healthcare outlet options.

## Results

### Socio-demographic and healthcare-seeking attributes of the participants

Slightly over half of the participants (52%) involved in the survey were male, 41% were aged 25–49, and 85% of the adults reported owning a phone. Household surveys revealed that most participants (81%) had sought some kind of treatment in the preceding 12 months (Table 1). Of these, 83% sought care from either public (53%) or private (30%) health facilities. The rest sought care from pharmacies/drug shops (8.5%), community health promoters (5.7%), or traditional healers (1.2%). Most of the participants (88.1%) were within less than 1 h of travel time to the nearest health facility, while the rest took between 1 and 2 h to reach the nearest facility, mostly using Boda Boda (motorcycle taxis) (47%) or walking (40%).

**Table 1:** Socio-demographic and healthcare-seeking attributes of the participants.

**Variable**	**Frequency**	**Percent**
**Gender**		
Male	135	52
Female	126	48
**Age**		
Children (1–17)	66	25
Young people (18–24)	49	18
Mid age (25–49)	109	41
Elderly (50+)	43	16
**Occupation**		
Farming	47	18
Boda boda	28	11
Fishing-related work	33	13
Mining-related work	28	11
Child/student	83	32
Other	40	15
**Adult phone ownership**		
Yes	166	85
No	29	15
**Sought treatment in the preceding 12 months**		
Yes	211	81
No	50	19
**Where treatment was sought**		
Public health facility	113	53
Private health facility	65	30
Traditional healer	3	1
Community health promoter	12	6
Drug shop/pharmacy	18	9
**Time to the nearest health facility**		
1–2 h	25	12
Less than 1 h	186	88
**Mode of transport to the nearest health facility**		
Vehicle	14	7
Walked	85	40
Motorcycle taxi	99	47

### Psychosocial factors

#### Community habits and norms

Though different by household, participants reported that usually, households with a sick member initially try home remedies that are immediately available and less costly. The prioritized home remedies include herbal medicine, leftover medications, and first aid interventions such as lowering fever with a wet cloth. After home remedies, the participants may then go for over-the-counter medications, initially purchasing painkillers and other fever-lowering medications, before they decide to seek care in health facilities, along with other interventions, such as visiting traditional and religious healers, especially if the illness persists in the first few days of treatment.

*We will start with home remedy, then to self-medication, then we will start looking for those healers because mostly even here in our community, you'll notice that a person will first do the first aid, then go to the hospital if it does not work…You'll notice that a person will first do the first aid, then go to the hospital if it does not work. If in three to four days the hospital does not give the required result, he/she will seek traditional healers to continue observing him/her*. (FGD-3 with rural women, Uriri)

#### Existence of Community Health Promoters (CHPs)

Some participants reported visiting CHPs (previously referred to as community health volunteers—CHVs) as part of their initial interventions before visiting health facilities. They observed that, lately, CHPs who live within their communities have been trained by the government and equipped with basic medications such as painkillers for initial interventions. In describing the convenience of CHPs in the communities, participants likened CHPs to a fire extinguisher in a factory that can be used before firefighters arrive.

…* If I'm close to a CHV, I can get first aid in time because they usually have Panadol, Septrin [painkillers]. They have drugs from the hospital. If they find that it is stomachache, they give me Flagyl for prevention until the following day. They are... the ones you first reach... You can be in a certain company, there is always a fire extinguisher. They are like that. In case there is a fire incident, then they are used to control the fire* (FGD-3 with rural women, Uriri).

#### Level of knowledge and awareness

The participants' level of knowledge and awareness about certain health conditions and how to handle them helped them in making healthcare-seeking decisions for their households. The specific knowledge factors reported to compromise household healthcare-seeking decisions included lack of ability to recognize diseases, stigma, and poor knowledge of medicine. For instance, the household head's level of awareness of disease etiology and symptoms determines where and when to seek care for a household member. Some participants reported that they always prioritize visiting health facilities because that is where they were told they would find experts to provide appropriate interventions.

*The reason as to why I go to the hospital and not any other way is because we are told that whenever we feel any changes in our body, the first thing to do should be to go to the doctor because he/she is the one who is skilled to know what is happening. You may go to someone else then you find yourself in more problems. That is why whenever I fall sick, I first seek medical assistance. He is the one who does all those to me and then advises me that “follow this way.” Then I follow* (IDI-9 with male miners, Masara).

### Enabling factors

#### Long waiting time

Participants described long waiting times as one of the main hindrances to seeking care, especially at public health facilities. The fear of spending an extended period waiting for treatment at the facilities, with the possibility of commodity stockout and being asked to purchase medications elsewhere, made people favor other interventions, including self-medication. While some people may understand if the long waiting time was due to large patient numbers, they were dismayed if the wait was caused by healthcare providers reporting late to duty or prioritizing their personal commitments over patient care.

*When you come here, you will stay for too long, and then after the lab test, you will be sent to the chemist to buy medication. So, I am taking the cheaper route, I just go to the chemist [pharmacy] direct I take the medicine, and then I go back home* (IDI-4 with female farmer, Uriri).

Participants emphasized the ability to tolerate a 2–3 h wait, preferably in the morning, to get treatment, but not the whole day due to other competing responsibilities and being dependent on daily wages, which they only earn when they work.

*You know, if it's a matter of coming to the clinic then I can try and go there very early in the morning. And if I can spend about 2 or 3 hours there then I go back, I can manage that but staying a whole day is not easy for me* (IDI-4 with female farmer, Uriri).

#### Remote residence and lack of transport

Participants reported residing in remote rural areas with poor road networks and lack of transport as reasons for the delay in accessing healthcare at public health facilities. Available means of transport, such as motorbike taxis, are withdrawn due to impassable roads or trucks when it rains or late at night, prompting the need for pragmatic decisions on where and when to seek healthcare.

*Even though I stay by the roadside, the means of transport such as a motorbike that you could use at that time is not available. That is why I'll take until morning when I can take the child to the hospital* (FGD-3 with rural women, Uriri).

#### Long distance to the health facility and lack of money

Related to the lack of transport and poor road networks, long distances to public health facilities and lack of funds to pay for transport and user fee charges sometimes drive the decision to go for cheaper alternatives, including traditional healers and self-medication through drug purchase from local pharmacies.

…* You understand that the hospital is far away and probably I don't have the money to take me to the hospital. For that reason, I normally opt to go to the chemist [pharmacy]. At the chemist [pharmacy], you can still go even if you only have 20 shillings [approx. 0.15 USD] and buy some Panadol. If the condition doesn't improve, then you can take the next action of going to the hospital* (IDI-7 with female miner, Masara).

#### Flexibility of service provisions

Another key factor cited in care-seeking decisions is having a favorite healthcare provider and flexibility in accessing services. For instance, a healthcare provider on duty can provide telemedicine to individuals with busy schedules, such as miners and fishermen, and connect them with CHPs promptly. This can dissuade households from relying on home remedies or self-medication, which may waste precious time from symptom onset to receiving a diagnosis and adequate treatment.

*I have a phone number of the people working at the clinic, particularly the nurse. I would just call the nurse using the number that I have; I will then go ahead and tell her my symptoms and ask whether a CHV can be sent to visit me* (IDI-7 with female miner, Masara).

#### Poor quality of service and stockouts

Perceived quality of services at public health facilities, including staff attitude and stockouts, is considered when households decide how to respond to an illness. If anticipating poor quality of care, especially stockout of prescribed medications, households explore cheaper and promising alternatives, including supernatural and self-medication interventions, before trying medication perceived as costly or healthcare perceived as inferior in quality.

*The first place… In fact, we used to come here at the health center, but nowadays, if you come here, you find that there are no drugs, no better treatment* (IDI-2 with male farmer, Uriri).

#### Being on health insurance

Participants reported that people with health insurance coverage, such as the National Health Insurance Fund (NHIF), now referred to as Social Health Insurance Fund (SHIF), often have several options available to them about where and when to seek care. The options available to them include accessing care at private health facilities where staff are friendly and have a better quality of care. However, it was pointed out that the cost of services in private health facilities is out of reach for households without health insurance, which require multiple payments for registration, tests, and medication, thereby indirectly discouraging visits. The cost of healthcare services acts as a critical enabler or barrier. As such, the possibility of sharing financial burdens within families or borrowing to afford healthcare or insurance makes healthcare decision-making for timely interventions a lot easier.

*So, with me I have the NHIF card, so I can just go to any hospital. For example, the New Global which is at Uriri Centre … So, you find that they charge a lot and the card can take care of that [which is better] than coming here then you are told that there are no drugs* (IDI-2 with male farmer).

### Need factors

#### Complexity and intensity of illness

The participants reported that the action taken depended on perceived complications and intensity of the illness and whether it is a known chronic disease that requires regular clinic visits. Home remedies, including leftover medications and over-the-counter medications from nearby pharmacies, are usually considered for conditions perceived to be mild and not serious.

*I already said that it depends on the illness. There are some infections that you do not go to the hospital and some that you have to go to the hospital. So, it depends on the disease you have (IDI-2 with female fisherfolk, Muhuru)*.

*You may end up wasting a whole day and maybe the pain you are feeling is not serious. So, it is better if I go there [pharmacy] and take medicine then go back. But if it's serious, then I will come here for a lab test which afterward they still send you to the chemist (IDI-4 with the female farmer, Uriri)*.

#### Depends on who in the household is ill

Participants reported that the action taken sometimes depended on who was ill within the household, with care being sought more quickly and from multiple sources for children, who are believed to be more vulnerable than adults.

*Now, if a child becomes sick and I feel a high fever, I run immediately to the nearby shops and then take painkillers that can relieve the fever. After controlling the fever, since I am near the hospital, I'll rush to the health facility* (FGD-3 with female farmers, Uriri).

#### Perceived etiology of the disease

Participants reported that where to seek care and the urgency depend on the perceived etiology of the disease. If the disease is perceived to be caused by supernatural powers, the focus is always to seek care from traditional or religious healers.

*It now depends on the type of disease someone is going through, like for ladies it is common like they feel* ‘*Rariw' (pain in their abdomen) … Then she is told go to so and so to give you medicine you drink … There are some [diseases] that people do believe cannot be treated in hospital like witchcraft, certain diseases that women suffer and so they look for the nearest person that can help them [with] the herbs* (FGD-4 with male living in urban Rongo).

## Discussion

In this study, we identified healthcare-seeking patterns in the context of emergent ill-health within households. We found that psychosocial (e.g., community norms), enabling (e.g., distance and availability of transport), and need (e.g., perceived severity and complexity of the illness) factors informed the household decision of whether, when, and where to seek care for ill members. We also found that, in most cases, households started their interventions by administering home remedies that included the use of leftover medications, local herbs, and wet clothes to control pain and fevers. Lack improvement made the intervention to be escalated to seeking care at public health facilities or from traditional and religious healers, which sometimes overlapped depending on the rate of patient recovery.

Overall, healthcare-seeking is a complex social process involving how symptoms are perceived, interpreted, and appraised to arrive at a decision that is often informed by people's ability and motivation to access care (31, 32). By understanding the patterns of household healthcare-seeking, public health providers can identify and address barriers to healthcare, including financial, cultural, and knowledge gaps, which may prevent timely and effective treatment (33). Synthesizing the challenges households encounter in care decision-making enables the design of targeted interventions that are culturally sensitive, accessible, and more likely to be owned and adopted by communities. Additionally, understanding the patterns of healthcare-seeking enables the improvement of health communication strategies between health facilities and communities, leading to early and appropriate care that prevents the spread of infections and mitigates the impact of chronic illnesses, thereby reducing the disease burden and improving overall community health outcomes.

Prioritizing home remedies postpones access to a qualified healthcare provider, resulting in diagnostic delays with far-reaching implications, especially for infectious diseases such as tuberculosis (TB) or diseases such as pneumonia and malaria in children and pregnant women (33, 34). Along with the decision of whether to seek help, the timing of that decision is critical (32) because delayed care-seeking presents complications in treatment with higher costs and poor prognosis (35, 36). For instance, Basharat et al. (35) demonstrated an increased cost of managing oral cancer due to delayed diagnosis occasioned by both patients and health facilities. Thus, as recommended by Ribeiro et al. (37), there is a need to significantly reduce diagnostic delays by identifying and considering behavioral patterns when designing measures to improve the delivery of health services.

Due to various reasons, including community habits and norms, cost and distance to health facilities, perceived severity of the illness, and the timing of symptom onset, people often explore interventions that are within reach and available, even if they are not necessarily optimal. For these communities, responsive healthcare interventions may acknowledge sequential healthcare-seeking behavior and work within this framework to introduce early points of professional intervention. Enhancing interventions such as those envisaged in Kenya's community health strategy, which recognizes the role of Community Health Promoters (CHPs), is likely to speed up the process of disease diagnosis and linkage to appropriate care (38–42). CHPs [formerly Community Health Volunteers/Workers (43)] are trained and equipped with basic diagnostic kits and medicines to handle common illnesses, including pneumonia and malaria, at the community level. The Kenyan government's strategy for CHPs is to integrate them into the primary healthcare system and reduce the burden on higher-tier healthcare facilities. The CHPs' conspicuous presence in the community as first responders can attract cases of ill-health for faster assessment and timely linkage to health facilities for specialized care, rather than wasting time on home remedies and self-medication with unknown efficacy. The CHPs can also be instrumental in promoting health literacy campaigns, government interventions such as registering for the new social health insurance fund, and educating households about the disease etiology, the importance of timely professional medical care, and the risks associated with delayed healthcare-seeking.

Health system contexts are key considerations for households when making decisions about seeking healthcare. Among the health system factors considered by households in our study were waiting times and stockouts at health facilities, which led households to opt for home remedies and self-medication. Other studies have also reported similar factors for poor access to or retention in chronic disease programs, especially at public health facilities (44–47). For instance, Liao et al. (48) in their study investigating the patient waiting experience for a medically underserved population at an outpatient surgical clinic, showed that patients spent over 68% of their time at health facilities waiting to see a provider, with whom they only spent 32% of the time at the clinic. This clearly indicates the need to address systemic and structural barriers within the healthcare system to improve access and utilization. One idea worth considering is streamlining operations at public health facilities, such as optimizing staff schedules, improving supply chain management to reduce stockouts, and enhancing flexibility in how services are offered, including facility opening and closing hours. Telehealth deployment for patients who do not need physical contact with the providers can reduce waiting times and enhance overall patient satisfaction. With this flexibility that results in improved services at health facilities, waiting time no longer becomes a variable that sways households toward making decisions to use home remedies or self-medication associated with delayed allopathic care.

This study has several limitations (1) we did not capture health providers' perspectives on health system-related challenges that preclude households from prioritizing seeking care at public health facilities. Considering the perspectives of healthcare providers would have provided a more complete picture of the context of healthcare in reference to health system resources and challenges. (2) We also acknowledge the challenge of social desirability and recall bias where participants may have given responses that conform with what they perceived to be right or were not able to remember clear how or the contexts they made certain healthcare seeking decision. (3) As an inherent constraint of qualitative studies, this work lacks generalizability and representativeness but we endeavored to achieve diversity in participant enrolment to make the findings applicable in similar settings elsewhere. Despite these limitations, this study still offers critical perspectives on factors that influence healthcare decision-making and the emerging patterns that are essential considerations for countries such as Kenya that are implementing universal health coverage.

In conclusion, Household healthcare-seeking in Migori County is shaped by intertwined psychosocial, enabling, and need factors that often delay timely access to formal care. Households first considered home remedies that included the use of leftover medications and local herbs, and control of pain before seeking care at public health facilities or from traditional and religious healers, with overlaps depending on the patient's recovery rate. Nurturing the success of the Kenyan revamped community health strategy with well-resourced and equipped CHPs is likely to minimize time to allopathic care by reducing the need for home remedies and self-medication. While strengthening health infrastructure remains critical, our findings suggest several additional policy directions. First, pharmacies and traditional or religious healers—who are frequently the first point of contact for households—could be integrated into a formal referral system through structured partnerships, training, and supervision to ensure timely linkage to biomedical care. Second, targeted financial mechanisms, such as low-cost micro-insurance schemes or conditional transport subsidies, could address financial and geographic barriers while improving equity in access. Third, expanding the role of Community Health Promoters to include patient navigation, health literacy, and referral follow-up would build trust and bridge gaps between households and facilities. By aligning health system reforms with the realities of household decision-making, we can accelerate earlier diagnosis, reduce preventable delays, and advance progress toward universal health coverage.

## Data Availability

The raw data supporting the conclusions of this article will be made available by the authors, without undue reservation.
